# Insight into functional microorganisms in wet–dry conversion to alleviate the toxicity of chromium fractions in red soil

**DOI:** 10.3389/fmicb.2022.977171

**Published:** 2022-08-10

**Authors:** Hongwei Liu, Ruiling Yuan, Emmanuel Konadu Sarkodie, Jiahui Tang, Luhua Jiang, Bo Miao, Xueduan Liu, Siyuan Zhang

**Affiliations:** ^1^School of Minerals Processing and Bioengineering, Central South University, Changsha, China; ^2^Key Laboratory of Biometallurgy of Ministry of Education, Changsha, China

**Keywords:** Cr pollution assessment, fraction distribution of Cr, wet and dry conditions, indicative functional microorganisms, red soil

## Abstract

Soil contamination with potentially toxic element such as chromium (Cr) poses a threat to the environment and human health. The environmental toxicity of Cr is related not only to the total Cr content but also to the distribution of Cr fractions. In this study, laboratory simulation experiments were conducted to explore the characteristics of Cr fractions and responses of the functional microbial community during dynamic leaching and static drying processes. The results showed that acid-soluble Cr and reducible Cr transformed into other relatively stable fractions under dry conditions, and ammonium nitrogen promoted the transformation. Nitrate-nitrogen was significantly positively correlated with Cr fractions in the wet stage (*p* < 0.05), while ammonium nitrogen showed the same relation in the dry process. Analysis of the microbial community showed that the bacterial and fungal genera Flavihumibacter, Altererythrobacter, Methylobacillus, Flavisolibacter, Lysobacter, and Cladosporium were related to the Cr fractions (acid-soluble Cr, reducible Cr, and oxidizable Cr) under wet conditions, while the microbial genera Ellin6067, MND1, and Ramlibacter were related to Cr fractions under dry conditions. Moreover, the proliferation of the functional microbial genera Methylobacillus, Ellin6067, and MND1 related to Cr fractions in the wet–dry conversion process alleviated the environmental toxicity of Cr. These findings provide useful information for the remediation of Cr-contaminated soils by monitoring the distribution fractions of Cr and the functional microbial community under wet–dry conditions.

## Introduction

Chromium (Cr) is an important industrial raw material and has been used widely in the chemical industry, for electroplating, and in leather tanning (Huang et al., 2009). Metallurgical and chemical industries discharge large amounts of Cr slag, which contains high concentrations of Cr (Rai et al., 2005; Hu et al., 2018). Due to the lack of appropriate disposal facilities and the high costs of Cr slag treatment, many illegally stacked Cr slag and abandoned Cr salt production sites have become potential sources of soil and groundwater pollution, which has attracted a great deal of public attention (Chen et al., 2016). Determination of the total Cr content is a useful method to evaluate the toxicity of Cr, but it provides minimal insight into the mobility of Cr in soils (Barajas-Aceves et al., 2007). The distribution and chemical association of potentially toxic elements in sediment and soil greatly affect their bioavailability and mobility (Pérez et al., 2008; Chen et al., 2017b; Jiang et al., 2020). Furthermore, studies have shown that the potential hazards of Cr under field conditions are dominated by its mobility and ecotoxicological properties in the solid–liquid phase (Shi et al., 2009; Zhang et al., 2020c). That is, it is not sufficient to focus only on the total amounts of potentially toxic elements; their mobility and bioavailability should also be examined. Sastre et al. (Sastre et al., 2004) reported that the fractions and proportion of potentially toxic elements in soil are key factors determining their environmental and ecological effects, including activity, bioavailability, toxicity, and migration characteristics. In addition, the bioavailability of Cr was shown to be significantly reduced when stabilizing and immobilizing agents were used to change the fractions of Cr (Barajas-Aceves et al., 2007; Chen et al., 2017a). Therefore, studies of the fraction transformation of Cr are important in environmental toxicity assessments and provide useful information for the remediation of Cr-contaminated soil.

As the most abundant organisms in nature that are highly sensitive to environmental variation, soil microorganisms are considered key indicators of environmental safety (Yan et al., 2020). Meanwhile, microbes are essential for biogeochemical cycling of elements. Growth and metabolism of microorganisms can lead to changes in physiochemical properties, such as pH, redox potential, and ionic strength of the soil. On the one hand, microbes affect different processes such as decomposing soil constituents as well as particle aggregation and influence soil texture and availability of nutrients. On the other hand, microbes directly transform the fraction of potentially toxic elements through mineralization and so on. For example, Bacillus megaterium transforms the fractions of Cr with higher activity into more stable fractions (Pradhan et al., 2019). The structure and function of bacterial and fungal communities in long-term Cr-contaminated soil have been recorded in detail using high-throughput sequencing methods (Fierer et al., 2012). There have been many studies regarding Cr and related microbial communities (Wang et al., 2009; He et al., 2016; Li et al., 2017a). For example, Desai et al. investigated the community shifts toward the dominance of Firmicutes from Proteobacteria in the long-term Cr-induced perturbation process (Desai et al., 2009). Hu et al. (2018) examined the direct toxicity of high concentrations of Cr to fungi that lead to changes in fungal communities, and exchangeable Cr and oxidizable Cr were shown to have marked effects on fungal communities. Pei et al. researched microbial community structure and function to indicate the severity of Cr contamination of the yellow river and found that potential indicator species, related to Cr such as Cr-remediation genera (Geobacter, PSB-M-3, Flavobacterium, and Methanosarcina) and the Cr-sensitive genera (Skermanella, Iamia, Arthrobacter, and Candidatus Nitrososphaera; Pei et al., 2018). Relative research revealed that the functional microbes Verrucomicrobia, Acidobacteria, and Planctomycetes positively related to Cr and secreted polysaccharides and proteins to detoxify Cr (Aceves et al., 2009). Accordingly, it is feasible to evaluate the toxicity of Cr in contaminated soil using related microorganisms and the occurrence fractions of Cr as the main indexes (Ma et al., 2007; Kasemodel et al., 2019). Moreover, the related microorganisms and the occurrence fractions can vary under dry and wet conditions (Barajas-Aceves et al., 2007; Aceves et al., 2009; Li et al., 2012).

The unique tropical and subtropical monsoon climate in southern China brings abundant rainfall and distinct fractions between the rainy and dry seasons (Cao et al., 2006). The formation of red soil in South China is related to the abundant rainfall (Huang et al., 2015; Wei et al., 2019). The red soil, accounting for approximately 11% of the total area of China, is an important soil resource in the tropical and subtropical regions of China (Li et al., 2007; Zhang et al., 2018). Moreover, the red soil in South China, where rice yield accounts for 80% of the national rice production, is generally characterized by high erosion risk and poor fertility (Xu et al., 2003; Yang et al., 2018). Unregulated Cr slag dumping and overuse of chemical fertilizers have resulted in the accumulation of Cr in red soil (Chen et al., 2010). Many groups have studied the spatial toxicity variability of Cr fractions under rainfall conditions (Aceves et al., 2009; Tan et al., 2015). However, there have been few studies of the migration and occurrence of Cr fractions in red soils in South China under dry and wet conditions (Han et al., 2001; Xu et al., 2013), which is essential for accurate environmental toxicity assessments and efficient remediation of soil pollution. In addition, as a green, efficient, and low-cost method, microbial remediation of Cr-contaminated soil is playing an increasingly important role in on-site remediation and has good prospects for further development. Consequently, understanding the function and structure of bacterial and fungal communities associated with Cr fractions under dry and wet conditions is essential for the assessment and bioremediation of Cr pollution in red soil.

In this study, we investigated the occurrence fractions of Cr and the corresponding effect on microbial structure and function under dry and wet conditions by simulation experiment of static and dynamic leaching of Cr slag. The main aims of this study were (i) to ascertain the vertical distribution of Cr fractions in soil profiles; (ii) to evaluate the effects of the Cr fractions on bacterial and fungal communities and the contribution of soil properties to the transformation of Cr fractions; and (iii) to determine the influence of the migration and fraction transformation of Cr on the dominant soil functional community. These findings will provide guidance for soil risk management and bioremediation of Cr pollution.

## Materials and methods

### Experimental design and sampling

Uncontaminated red soil was collected from Yuelu County, Changsha City, Hunan Province, China (E112°54′30″, N28°10′35″N). A total of 28 samples were taken at four sites selected at random from a depth of 1–2 m. Cr slag samples were collected from an abandoned Cr plant located in Changsha City, China (E112°58′0″, N28°16′23″). The obtained soil and Cr slag were gathered in polyethylene bags and transported immediately to the lab. The samples were divided into three portions after removing plant remains and stones. The first portion was stored at −80°C for molecular analysis, the second was stored at 4°C for examination of soil characteristics, and the third was air-dried and passed through 0.84-mm sieves for the simulation experiments.

The laboratory simulation experiments of Cr slag-contaminated soil under dry and wet conditions were performed to investigate the occurrence of Cr and its effects on microbial function and structure. Deionized water was added to adjust the soil water content, then the samples were mixed well and passed through a 0.5-cm sieve. The uniform mixture of original soil was added into six polymethylmethacrylate (PMMA) columns. Each column was divided into four separate chambers (diameter 20 cm, height 100 cm). Red soils with the same height on PMMA columns were analyzed. The control group (CK) and treatment group (TG) were distinguished by non-stacking or stacking of Cr slag, respectively. For the TG, Cr slag with a height of 10 cm was evenly loaded onto the soil columns (error < 1.0 mm). Cr slag and soil samples were separated using double gauze. All PMMA columns were wrapped with tinfoil to avoid the effects of sunlight. In our previous study, spraying 205 ml of water per week saturated the soil layer at 90 days, and the acid-soluble and reducible Cr solutions migrated downward (Zhang et al., 2020c). To mimic the leaching process (LP), the CK and TG groups were sprinkled with water weekly using a spray kettle and sampled on days 0 and 90. In addition, to explore the occurrence fractions of Cr in the wet and dry stages, samples were taken at 180 days after natural drying (ND) for 90 days in PMMA columns. The stacked Cr slag was completely removed at each sampling time point, and soil samples were taken after marking 20 cm on the cross-section of the PMMA column. Samples were taken from different chambers at three biological replicates. The stacked Cr slag layer samples were used as the G layer. The soil profile was defined as Top (T layer, 0–20 cm), Middle (M layer, 20–40 cm), and Substratum (S layer, 40–60 cm).

### Soil physiochemical properties and Cr slag determination

The samples used for the determination of soil physiochemical properties were air-dried rapidly and screened with 200-mesh sieves. The soil pH and oxidation–reduction potential (ORP) were determined in deionized water extract (soil-to-water ratio 1:2.5, wt./vol.) using a pH meter with a glass electrode and Ag/AgCl reference electrode (BPH–220; Bell Instrument Equipment Co. Ltd., Dalian, China; Hao et al., 2019). The total content of potentially toxic elements was determined according to the method of Liu et al. (2016). Briefly, about 0.500 g of soil or Cr slag sample was digested with a mixture of HNO_3_/HF/HClO_4_ (10:5:2, vol./vol./vol.) on an electric heating plate (XJS20-42; Laboratory Instrument Equipment Co. Ltd., Tianjin, China) and the concentrations of metal(loid)s (Cr, Fe, K, Al, Mn, Ca, As, Na, and Mg) in the digested fluid samples were measured using inductively coupled plasma optical emission spectroscopy (ICP-OES; Optima 5300DV; PerkinElmer, Shelton, CT, United States; Liu et al., 2016). According to a modified three-step sequential extraction method of the European Community Bureau of Reference (Rauret et al., 1999), the four fractions of Cr in soil samples, i.e., acid-soluble fraction (F1), reducible fraction (F2), oxidizable fraction (F3), and residual fraction (F4), were extracted (Deng et al., 2019). Furthermore, the contents of ammonium nitrogen (NH_3_-N) and nitrate-nitrogen (NO_3_-N) were determined using the indophenol blue method and calcium chloride method, respectively (Lu, 1999). The soil organic matter (OM) content was measured using a total organic carbon analyzer (BOCS301; Shimadzu, Kyoto, Japan; Deng et al., 2019). The soil total potassium (TK), total phosphorus (TP), and total nitrogen (TN) were determined using ICP-OES (Optima 5300DV; PerkinElmer), ammonium molybdate spectrophotometry, and the semi-micro-Kjeldahl procedure, respectively (Deng et al., 2017). In addition, the available potassium (OK) and available phosphorus (OP) were analyzed using flame photometry and spectrophotometry, as described previously (Song et al., 2018). The soil characteristics are listed in Supplementary Table S1.

### DNA extraction and high-throughput sequencing

Total genomic DNA was extracted from soil and Cr slag samples by Guangdong Magi Gene Biotechnology Co., Ltd. (Guangzhou, China) using E.Z.N.A. Soil DNA kits (Omega Bio-Tek, Norcross, GA, United States) in accordance with the manufacturer’s instructions. Each sample was processed in triplicate. DNA concentration and quality were determined using a NanoDrop2000 Spectrophotometer (Thermo Scientific, United States). Sequencing libraries were constructed using NEBNext® Ultra™ DNA Library Prep Kits for Illumina® (New England Biolabs, Ipswich, MA, United States). The libraries were sequenced on an Illumina Hiseq X-Ten platform with 150-bp paired-end reads. The Btrim program was used to trim reads with quality scores <20 (Kong, 2011) and the FLASH program merged forward and reverse reads of the same sequence (Magoc and Salzberg, 2011). The trimmed FASTQ data were converted to FASTA format, and UPARSE was used to cluster sequences with 97% identity to the same operational taxonomic unit (OTU; Edgar, 2013). Taxonomic assignment with a minimal 50% confidence score was carried out using RDP Classifier.^1^ Microbial sequencing data were submitted to the NCBI Sequence Read Archive (SRA) database with the accession numbers SRP299378 and SRP299488.

### Data processing and statistical analysis

For microbial community analysis (Li et al., 2017b), α-diversity indexes (including Shannon diversity, Simpson_evenness, and observed_species), principal coordinate analysis (PCoA), and redundancy analysis (RDA) were performed using the base R package vegan (version 3.7.1). RDA was carried out using Canoco 5 software to probe the correlations between selected microbial genera and soil characteristics. Venn diagrams were plotted using the Venn Diagram R package. Statistical significance of the differentially abundant genera was examined by Statistical Analysis of Metagenomics Profiles (STAMP; Parks et al., 2014), and Pearson’s correlation coefficients were calculated with Minitab version 17.0 (Minitab Inc., State College, PA, United States). The correlation coefficients were displayed on heat maps using the heatmap R package. The linear discriminant analysis effect size (LEfSe) was analyzed to detect the biomarkers in environmental samples using the online interface Galaxy.^2^ One-way analysis of variance was performed to determine the differences in parameters (e.g., soil characteristics, potentially toxic element contents, and α-diversity indexes). Generally, the least significant difference test was used to calculate the significance of differences between means (*p* < 0.05). The PICRUSt2 tool predicted the metagenome based on the OTUs of 16S rRNA sequence data sets (Douglas et al., 2020). The predicted metagenomes were functionally annotated by Kyoto Encyclopedia of Genes and Genomes (KEGG) pathway analysis. STAMP was used to evaluate the significance of differences in metagenomic profiles and to visualize the categorized metagenomes generated by PICRUSt2. The FUNGuild website^3^ was used to predict the ecological guilds of fungi present in the samples based on OTUs of internal transcribed spacer (ITS) rRNA sequence data sets (Nguyen et al., 2016).

## Results

### Effects of wet and dry conditions on Cr fractions in Cr slag and red soil

**Figure 1:**
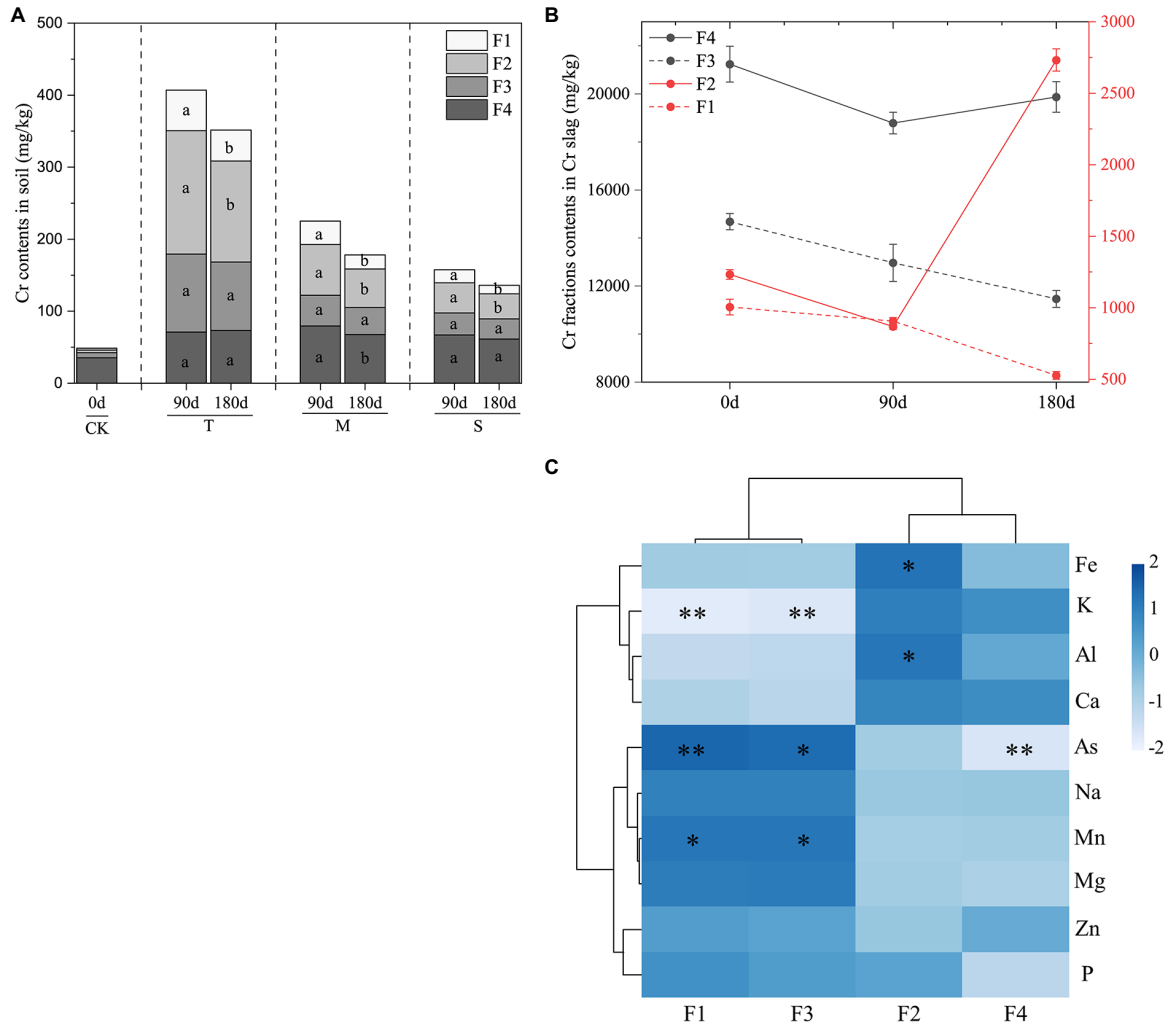
**(A)** The content of four chromium (Cr) fractions in soil. **(B)** The contents of each Cr fraction in the Cr slag layer. **(C)** Correlation analysis of element content and Cr fractions in Cr slag. Data are presented as means ± SD (*n* = 3). Different lowercase letters above the bars indicate significant difference (*p* < 0.05, LSD) among different groups. CK, control group; T, top layer; M, middle layer; S, substratum layer; F1, acid-soluble Cr; F2, reducible Cr; F3, oxidizable Cr; F4, residual Cr. The value of *p* < 0.05 is marked with “*” and *p* < 0.01 is marked with “**.”

As shown in Figure 1A, the concentrations of the four fractions of Cr were markedly increased in the TG compared with the CK. In the TG, the proportions of F4 and F3 decreased from 58.2 to 15.1% and 28.6 to 19.9%, respectively, while the proportions of F1 and F2 increased from 6.3 to 15.9% and 8.5 to 42.3%, respectively. During the 90-day LP, the contents of F1 and F2 decreased in the T layer but increased in the M and S layers over time. The contents of F1 and F2 decreased in all layers during 90 days of ND compared with the LP. In comparison, the total Cr content of the ND treatment group was significantly lower than that of the LP treatment group. Furthermore, the contents of F3 and F4 remained constant under wet and dry conditions.

The results of fraction analysis of Cr slag samples are shown in Figure 1B. The total Cr content of Cr slag on day 0 was higher than those on days 90 and 180. However, the distributions of Cr fractions differed markedly between the LP and ND treatments. In the LP stage, the contents of F1, F2, F3, and F4 decreased by 9.67, 29.57, 11.70, and 11.52%, respectively. Nevertheless, there was a major shift in the ND stage. Compared with the LP, the contents of F2 and F4 increased by 68.23 and 5.47%, respectively, while the contents of F1 and F3 decreased by 41.96 and 11.56%, respectively, in the ND stage. The correlations between the four Cr fractions and the metal(loid)s in the Cr slag were determined by analyzing the elemental contents of Cr slag in the G layer (Figure 1C). The contents of metal(loid)s in Cr slag played important roles in the effects of the treatment processes. F1 and F3 were positively correlated with As and Mn, but negatively correlated with K. The correlations between Fe, Al, and F2 were highly positive.

### Relationships between Cr fractions and soil properties under wet and dry conditions

Soil properties, including pH, OM, ORP, TN, TP, TK, NH_3_-N, NO_3_-N, OP, and OK, were analyzed (Supplementary Table S1). The results showed that the occurrence of Cr fractions affected soil properties, which changed under wet and dry conditions. The OM and NO_3_-N contents decreased by 60 and 45.7%, respectively, in the G layer at the LP stage, and also decreased significantly in different soil layers. The OM and NO_3_-N contents were roughly constant in the ND stage. Meanwhile, TN, TP, NH_3_-N, TK, and OK differed significantly between the LP and ND treatments. The pH and ORP also played important roles in the occurrence of fractions of Cr in soil. In contrast to the CK, pH increased by 0.2–0.5 units and 0.1–0.4 units in the LP and ND stages and ORP decreased by 7–18 mV and 4–8 mV in the LP and ND stages, respectively. The relationships between the four fractions of Cr and soil characteristics were examined using Pearson’s correlation analysis (Table 1). In the wet–dry conversion process, the correlation results showed that F1, F2, and F3 were markedly positively correlated with TN and pH, while F4 was significantly positively correlated with NH_3_-N (*p* < 0.05). Moreover, F1, F2, and F3 were significantly positive correlated with NO_3_-N at the LP stage but with NH_3_-N at the ND stage.

**Table 1:** Relationships between four fractions of Cr and soil properties were also shown by Pearson’s correlation analysis.

Time	Fractions	Properties									
		OM	TP	OP	TN	NH_3_–N	NO_3_–N	TK	OK	pH	ORP
90 days	F1	n.s.	n.s.	n.s.	0.68*	n.s.	0.705*	n.s.	n.s.	0.807**	n.s.
	F2	n.s.	n.s.	n.s.	0.718*	n.s.	0.705*	n.s.	n.s.	0.817**	n.s.
	F3	n.s.	n.s.	n.s.	0.727*	n.s.	0.709*	n.s.	n.s.	0.806**	n.s.
	F4	n.s.	n.s.	n.s.	n.s.	0.86**	n.s.	n.s.	n.s.	n.s.	n.s.
180 days	F1	n.s.	n.s.	n.s.	0.699*	0.836**	n.s.	n.s.	n.s.	0.861**	n.s.
	F2	n.s.	n.s.	n.s.	0.686*	0.807**	n.s.	n.s.	n.s.	0.873**	n.s.
	F3	n.s.	n.s.	n.s.	0.689*	0.79*	0.672*	n.s.	n.s.	0.886***	n.s.
	F4	n.s.	n.s.	n.s.	n.s.	0.8**	n.s.	n.s.	n.s.	n.s.	n.s.

F1, acid-soluble Cr; F2, reducible Cr; F3, oxidizable Cr; F4, residual Cr. The value of *p* < 0.05 is marked with “*,” *p* < 0.01 is marked with “**,” and *p* < 0.001 is marked with “***.” n.s. representation is not significant.

### Diversity, composition, and abundance of microbial community

A total of 1,717,013 high-quality 16S rRNA gene reads and 3,207,913 high-quality ITS rRNA gene sequence reads were obtained from the high-throughput sequencing under wet and dry conditions. With Cr migration, the α-diversity results showed that the richness (observed species) and diversity (Shannon and Pielou’s evenness) were not significantly different (*p* > 0.05) between different layers of soil (Supplementary Table S2). The temporal distribution of changes in the microbial diversity of the whole soil (0–60 cm) was then determined. Compared with ND, the α-diversity showed that the richness of bacteria increased and the diversity decreased significantly during the LP; however, the α-diversity results of fungi showed the converse trend (Supplementary Table S3).

**Figure 2:**
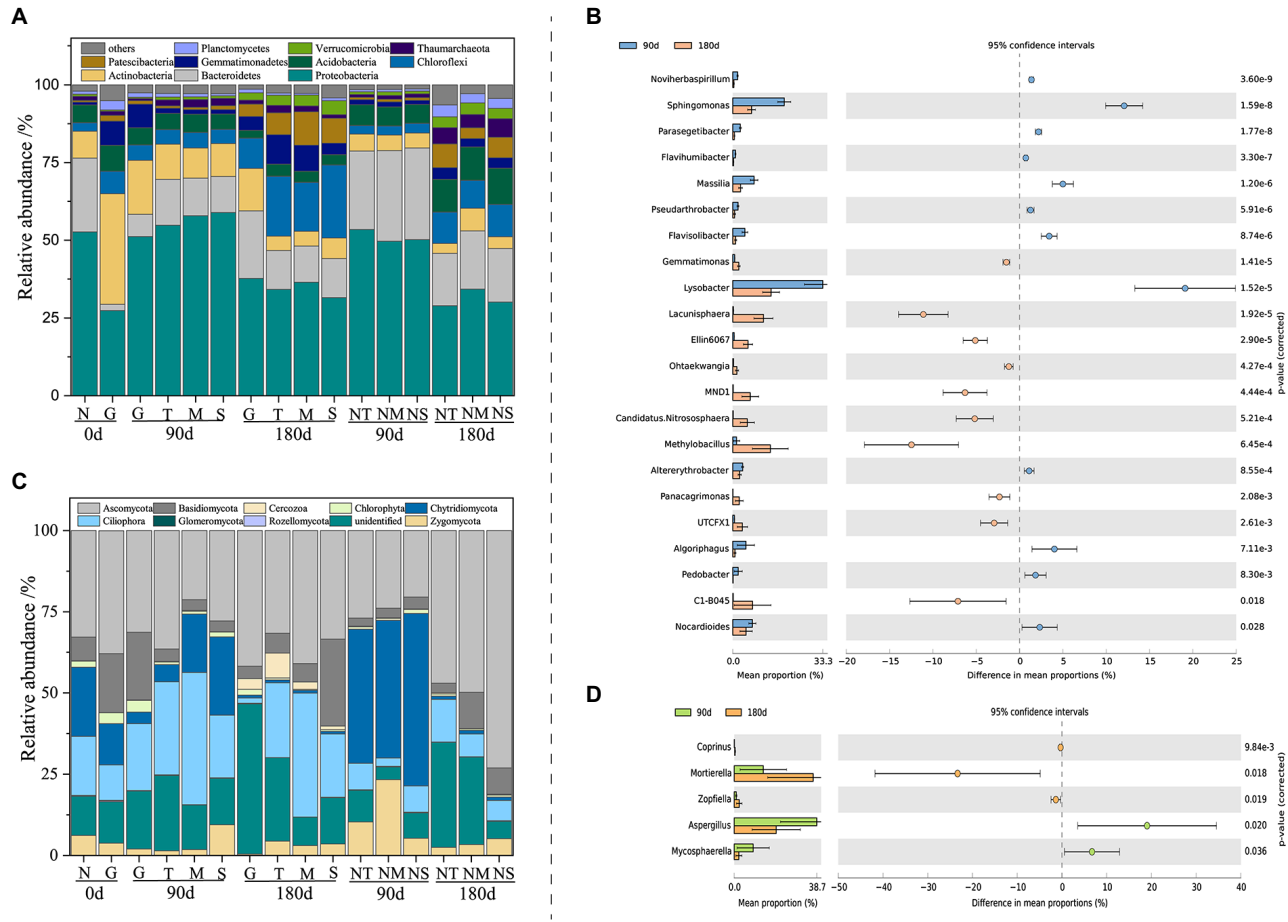
Relative abundance of bacterial **(A)** and fungal **(C)** phyla in different soil samples. The differentially abundant of bacterial **(B)** and fungal **(D)** genera between 90-day and 180-day treatment (*n* = 3). N, control group; T, top layer; NT, top layer of control group; M, middle layer; NM, middle layer of control group; S, substratum layer; NS, substratum layer of control group; G, Cr slag layer.

The results of PCoA showed that the microbial community structures of both bacteria (analysis of similarities (ANOSIM), *R* = 0.9024, *p* = 0.001) and fungi (ANOSIM, *R* = 0.8139, p = 0.001) exhibited temporal succession patterns between the wet and dry processes (Supplementary Figures S1A,B). Venn diagrams (Supplementary Figures S1C,D) revealed 1,175 and 561 core OTUs between bacterial and fungal communities, and there were more unique OTUs during the LP compared to ND. This suggested that the diversity of microbial species composition was higher in the LP than in the ND treatment. To clarify the microbial compositions at the LP and ND stages, the dominant phyla and genera of bacteria (Figures 2A,B) and fungi (Figures 2C,D) were compared. The bacterial phyla Proteobacteria, Actinobacteria, Bacteroidetes, Acidobacteria, Gemmatimonadetes, and Chloroflexi and the fungal phyla Ascomycota, Zygomycota, Ciliophora, and Chytridiomycota were dominant in the soil of the CK group. However, the relative abundances of Proteobacteria, Actinobacteria, Chloroflexi, Ciliophora, and Ascomycota increased, while the relative abundances of Bacteroidetes, Acidobacteria, Zygomycota, and Chytridiomycota decreased, in the LP stage. In the ND stage, the relative abundances of Chloroflexi, Gemmatimonadetes, Ciliophora, and Cercozoa increased, while those of Bacteroidetes, Acidobacteria, Chlorophyta, and Ascomycota decreased. Furthermore, at the genus level, bacteria and fungi differed significantly between the LP and ND stages (*p* < 0.05; Figures 2B,D). LEfSe, as a reliable analytical method for examining biomarkers in the microbiome (Yang et al., 2020), further confirmed that the keystone taxa of the bacterial community were significantly more abundant in LP than ND soil samples (Supplementary Figure S2). Moreover, we did not find biomarkers of fungi.

### Effects of Cr fractions and other environmental factors on the microbial community

**Figure 3:**
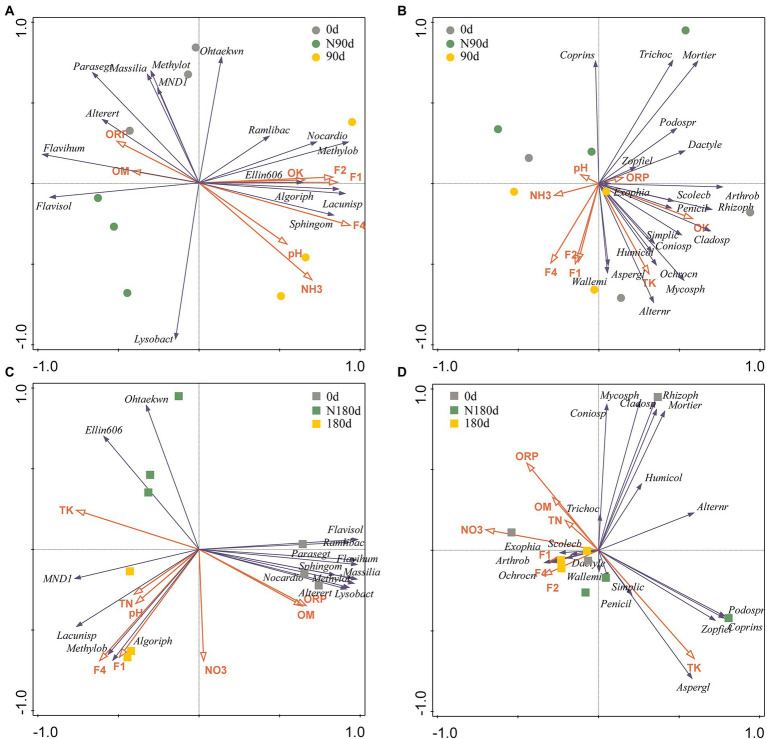
The ordination plot from redundancy analysis (RDA) showing the relationship between the microbial community structures and environmental factors. **(A)** Bacterial genera with more than 2% abundance of 90 days; **(B)** the fungal genera with more than 2% abundance of 90 days; **(C)** the bacterial genera with more than 2% abundance of 180 days; **(D)** the fungal genera with more than 2% abundance of 180 days. Each point represents the individual microbial community in soils. Arrow direction indicates the correlation among soil properties; arrow length indicates the strength of the correlation. ORP, oxidation–reduction potential; TN, total N; NH_3_, ammonium nitrogen; NO_3_, Nitrate nitrogen; TK, total K; OK, available K; OM, organic matter.

The results shown in Supplementary Figure S3 indicate that the environmental parameters, such as TK, OK, ORP, pH, NO_3_-N, NH_3_-N, F1, F2, F3, and F4, influenced the composition and abundance of the microbial community (0.001 < *p* < 0.01, Monte Carlo permutation test). Among these factors, NO_3_-N, ORP, and pH corresponded more to the LP microbial community, while other environmental factors were more strongly related to the microbial community in the ND sample. RDA was conducted to further assess the relationships between selected soil properties and bacterial and fungal genera with relative abundances >2% (Figures 3A–D). In the bacterial community of the LP samples, the contributions of F4 and NH_3_-N followed the trend F4 (explained 51.70%, *p* = 0.002) > NH_3_-N (explained 19.10%, *p* = 0.034). Furthermore, in the fungal community of the LP sample, the contributions of F4, OK, and TK followed the trend F4 (explained 33.30%, *p* = 0.01) > OK (explained 25.20%, *p* = 0.011) > TK (explained 15.10%, *p* = 0.042). In the bacterial community of the ND sample, the contributions of TK and TN followed the trend TK (explained 54.80%, *p* = 0.006) > TN (explained 20.10%, *p* = 0.03). Moreover, in the fungal community of the ND samples, the contributions of NO_3_-N and TK followed the trend NO_3_-N (explained 33.60%, *p* = 0.048) > TK (explained 16.60%, *p* = 0.011). Thus, these selected environmental factors (F4, TK, OK, TN, NH_3_-N, and NO_3_-N) were vital factors affecting the bacterial and fungal communities in the LP and ND stages.

**Figure 4:**
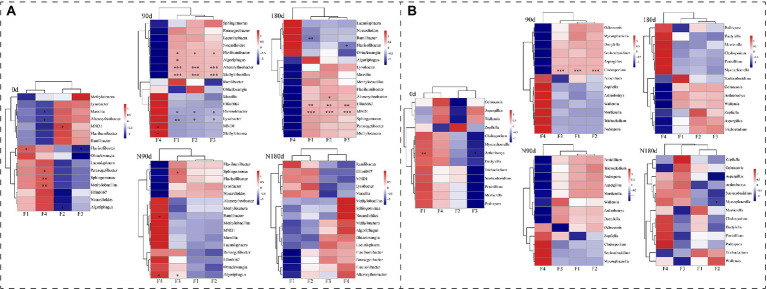
Correlation heat map of the relative abundance >2% bacterial genera **(A)**, fungal genera **(B)**, and four fractions of Cr in soil. X- and Y-axis are four fractions of Cr and genera, respectively. *R* in different colors to show, the right side of the legend is the color range of different *r* values. The value of *p* < 0.05 is marked with “*,” *p* < 0.01 is marked with “**,” and *p* < 0.001 is marked with “***.”

Heat map analyses were performed to correlate bacterial and fungal genera with relative abundances >2% and four Cr fractions (Figures 4A,B). Within the LP treatment bacterial community, Flavihumibacter, Altererythrobacter, and Methylobacillus were positively related and Flavisolibacter and Lysobacter were negatively correlated with F1, F2, and F3 (*p* < 0.05). In addition, Algoriphagus was positively correlated with F1 (*r* = 0.671, *p* = 0.047). In the ND treatment bacterial community, Ellin6067 and MND1 were positively related to F1, F2, and F3, while Ramlibacter was negatively correlated with F1 (*r* = −0.707, *p* = 0.01). In the LP treatment fungal community, Cladosporium was positively correlated with F1 (*r* = 0.947, *p* = 0), F2 (*r* = 0.94, *p* = 0), and F3 (*r* = 0.949, *p* = 0.001). These results showed that the variations of Cr fractions in LP and ND treatments significantly altered the associated microbial communities.

## Discussion

### Effects of dry and wet conditions on the stabilization of Cr fractions in soil

The changes in dry and wet conditions altered the occurrence of Cr in soil (Figure 1A). During the LP stage, the contents of F1 and F2 decreased in the T layer and increased in the M and S layers. After 90 days of ND, the contents of F1 and F2 decreased in all soil layers. This may have been due to the water saturation of the soil profile causing reductions in the contents of F1 and F2 in the T layer in the static dry state, while those in the M and S layers increased during the LP and decreased during ND. Furthermore, the proportions of F1 and F2 reduction in the ND stage were not the same in each layer. The results showed that the four fractions of Cr could be transformed into one another over time (Jalali and Khanlari, 2008), and F1 and F2, which readily migrate, could change into other relatively stable fractions under dry soil conditions. As documented in the literature, most of the Cr fractions that readily migrated are bioavailable. In addition, the stable occurrence fractions of Cr in soil greatly reduced the environmental toxicity (Barajas-Aceves et al., 2007; Pérez et al., 2008). These results indicated that the environmental toxicity of Cr was restricted by soil water content (Cheng et al., 2014). Shaheen et al. (2014) concluded that the dynamic migration of Cr was related to the duration of soil exposure to floods, because drivers of Cr element mobility required a certain amount of time to provoke reactions under shifting conditions. Soil Cr pollution in the experiment was caused by the leaching of Cr slag. However, the occurrence fractions and transformation of Cr slag as the sources of soil Cr pollution remained unclear. To further explore the occurrence and transformation mechanisms of Cr fractions under wet and dry conditions, the contents of four fractions of Cr slag at different time points were analyzed (Figure 1B). The results indicated that the contents of the four fractions in Cr slag diminished during the 0–90-day LP. In our previous study, F1 and F2 migrated downward in the soil profile (Zhang et al., 2020c). It was suggested that the decreases in F3 and F4 may have been due to the shifts of F3 and F4 to the fractions that migrated easily. Moreover, the contents of F1 and F3 were reduced, while the contents of F2 and F4, were increased under ND conditions. To investigate the relationships of these variations, the correlations between metal(loid)s in Cr slag and four Cr fractions were analyzed (Figure 1C). The literature shows that the contents of Fe, Mn, and Al in Cr-contaminated soil are significantly enriched with increasing monsoon precipitation. The accumulation of different fractions of Cr was mainly due to the flocculation and coagulation of Fe/Mn oxyhydroxides (Papassiopi et al., 2009; Koukina and Lobus, 2020). However, some studies found no significant correlations between the total concentrations of Cr and As in soil (Huang et al., 2010; Tu et al., 2011). Meanwhile, there was a lack of evidence for the mutual restriction of K and Cr fractions. The drying conditions have been shown to have a greater influence on the stabilization of Cr fractions, but the related elements of Cr fraction transformation require further verification.

### Effects of nitrogen on Cr fractions and microbial community in wet–dry stages

F1 and F2, which migrate readily, were associated with NO_3_-N in the LP stage but positively correlated with the NH_3_-N content in the ND stage. The content of NH_3_-N significantly increased by 63.48% and decreased by 18.6%, and NO_3_-N increased slightly by 12% and decreased by 7.5% in the LP and ND stage. It has been reported that Cr pollution in soil stimulates denitrification activity and ammonification, and inhibits nitrification (Ye et al., 2005). The exchangeable form of Cr was transformed into a strongly bound form during the drought-rewetting process, and the differences between NH_3_-N and NO_3_-N play important roles in this process (Lin et al., 2018). Previous studies showed that loosely bound F1 and F2 were converted to more stable fractions in the drying stage. Therefore, the differences of soil in dry and wet environments caused the changes in the correlation between the nitrogen cycle and Cr fractions, and NH_3_-N actively promoted the transformation of loosely bound F1 and F2 into other more stable fractions.

The fractions transformation of Cr during wet–dry conversion was significantly related to nitrate nitrogen and ammonium nitrogen in soil. That is to say, nitrogen cycling-related microorganisms should be particularly concerned. According to relative research, Gemmatimonadetes devoted to denitrification can drive nitrogen cycle and provide electron acceptors in soils and other environments (Diamond et al., 2019; Zhang et al., 2021a,b). Specifically, Planctomycetes, and Acidobacteria genomes have been reported encode utilization of various inorganic and organic nitrogen sources, nitrification and denitrification, and transport of iron and potentially toxic elements (Diamond et al., 2019). In addition, heavy–metal–resistant bacteria Gemmatimonadetes and Acidobacteria involved in C/N cycling and effectively promoted denitrification and ammonification in potentially toxic elements contaminated soils, while Planctomycetes plays an important role in nitrification (Diamond et al., 2019; Zhang et al., 2021a,b). Compared to CK group, the relative abundances of Acidobacteria decreased in the LP stage. In the ND stage, the relative abundances of Gemmatimonadetes increased, while Acidobacteria decreased. Overall, the abundance of these two genera increased. Unfortunately, Planctomycetes was not detected in two stages. It has been reported that the main contributor to the change of nitrogen content in Cr contaminated soil is the change of bacterial community (Zhang et al., 2021a,b). In a word, the variation trend of content NH_3_-N and NO_3_-N in the nitrogen cycle was consistent with the changes in microorganisms associated with nitrogen metabolism and the function of microbial community. Soil microorganisms have been used as important indexes to evaluate potentially toxic element pollution and play crucial roles in the cycling and distribution of nitrogen in the dry and wet stages (Zhang et al., 2020b). As shown in Figure 3, the significant differences in contents of nitrogen (TN, NO_3_-N, and NH_3_-N) contributed markedly to the changes in Cr fractions and microbial communities. Changes were detected in nitrogen in Cr slag, and the contents of NH_3_-N and NO_3_-N in the LP and ND stages decreased significantly (Supplementary Table S1). Therefore, we speculated that the transformation of nitrogen in soil was partly due to the downward migration of Cr slag, with the remainder due to nitrogen metabolism by microorganisms. It has been reported that the microbial communities in soils are markedly influenced by high contents of Cr and organic N (Kong et al., 2019). Moreover, He et al. suggested that land use shifts markedly affected the bacterial processes involved in nitrogen cycling, and the nitrifying bacteria, Nitrosospira and Nitrospira, were markedly more abundant in woodland and arable land than in natural wetland (He et al., 2020). We showed that the abundance of nitrogen metabolism-related genes was significantly higher in the LP than in the ND stage (*p* < 0.05; Supplementary Figure S4). The circulation of nitrogen in dry and wet soil affected the occurrence fractions and transformation of Cr and the distribution of bacteria and fungi communities. Nitrogen plays an important role in the occurrence fractions of Cr and the distribution of the microbial community in Cr-contaminated soil; therefore, attention should be paid to the treatment of Cr pollution in the future.

### Effects of dry and wet conditions on the microbial community related to Cr fractions

To identify how the occurrence fractions and transformation of Cr affected the soil microbial composition, we performed heat map analyses to correlate bacterial and fungal genera with relative abundances >2% and the four Cr fractions (Figures 4A,B). Several studies reported that microbial strains were restrained by the presence of potentially toxic elements, and some genera, such as Flavihumibacter, Ramlibacter, Lysobacter, and Flavisolibacter, were resistant to Cr and were biomarkers of Cr-contaminated soil (Garcia-Gonzalo et al., 2017; AN et al., 2018; Jia et al., 2019). This was due to NADH-dependent Cr reductase in the membrane and protoplast, which could reduce the toxicity of Cr (Ramírez-Ramírez et al., 2004). However, the correlations between Cr fractions and bacterial communities, especially fungal communities, were still unclear in the dry and wet stages. In the LP treatment bacterial community, Flavihumibacter, Altererythrobacter, Methylobacillus, Flavisolibacter, and Lysobacter were related to F1, F2, and F3 (*p* < 0.05). In addition, in the ND treatment bacterial community, Ellin6067 and MND1 were positively related to F1, F2, and F3, and Ramlibacter was negatively correlated with F1. In the LP treatment fungal community, Cladosporium was positively correlated with F1, F2, and F3. Several studies showed that Cladosporium biomass identified from Cr-contaminated soil could adsorb and reduce Cr (VI) and thus decrease its environmental toxicity (Batista et al., 2016; Garza-Gonzalez et al., 2017). In the ND treatment fungal community, there were no significant correlations between the four Cr fractions and the selected fungal genera. As shown in Figure 2; Supplementary Figures S1, S2, the migration and occurrence of Cr fractions under dry and wet conditions altered the soil microbial communities. The microbial diversity was higher in the LP than the ND stage. In addition, at the phylum and genus levels, bacteria and fungi differed significantly between the LP and ND stages (*p* < 0.05). Similar results in previous studies (Sheeba et al., 2017; Kasemodel et al., 2019) showed that high concentrations of Cr altered the diversity of soil bacteria and archaea between dry and wet seasons. Hu et al. (2018) also reported that long-term Cr pollution caused marked shifts in soil physicochemical properties, and a considerable portion of Cr fractions of exchangeable Cr had the highest impact on fungal communities. Therefore, understanding the distinction between bacterial and fungal strains associated with Cr fractions in dry and wet conditions will play an important role in improving the accuracy of soil Cr pollution assessment. In addition, it will be necessary to screen for related microbial species and explore the mechanisms underlying their changes in future studies.

### Effects of Cr fractions on microbial community function

**Figure 5:**
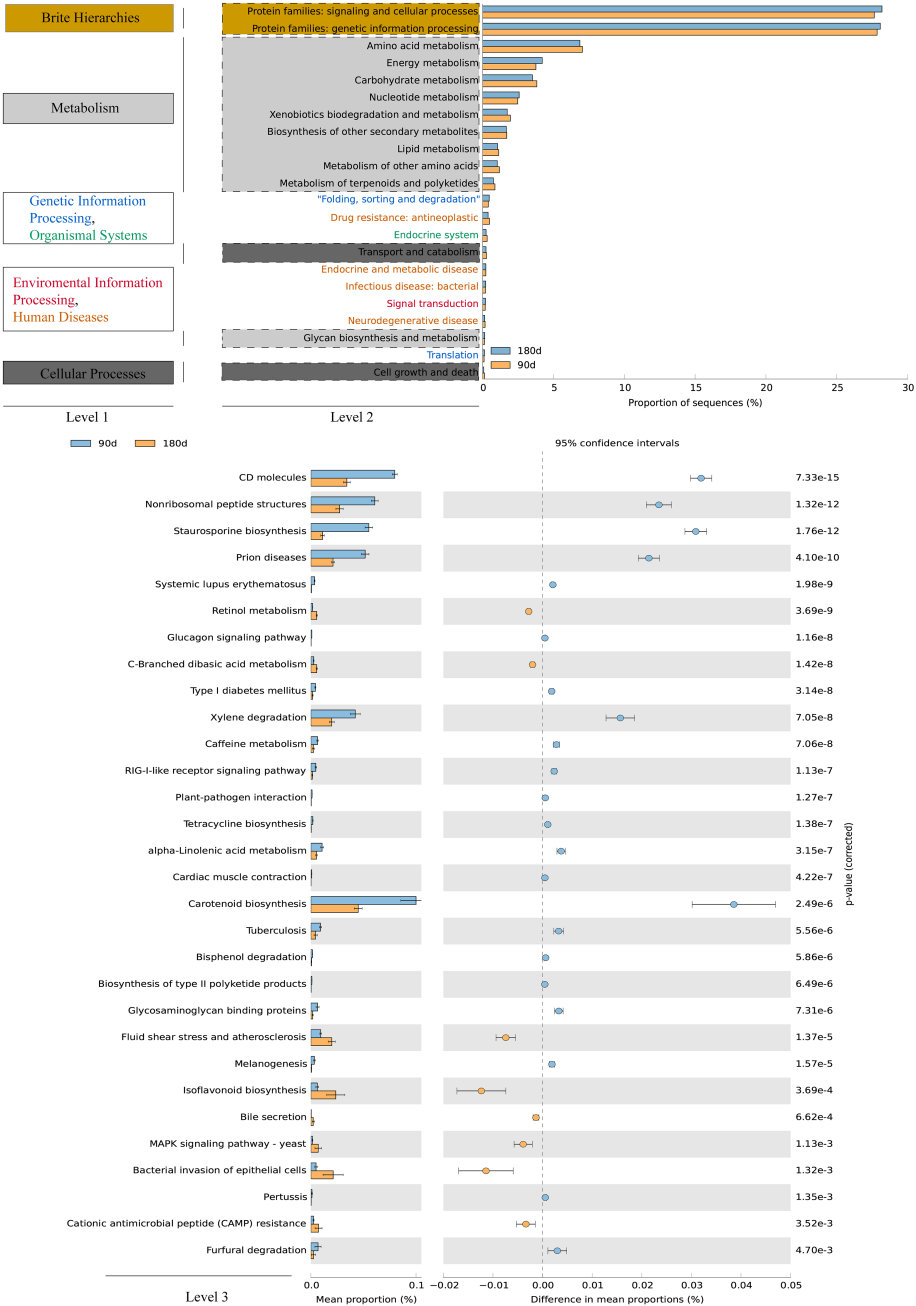
Variations in bacterial functional profiles between 90-day and 180-day treatment were annotated using PICRUSt2 (functional categories), following Welch’s *t*-test.

**Figure 6:**
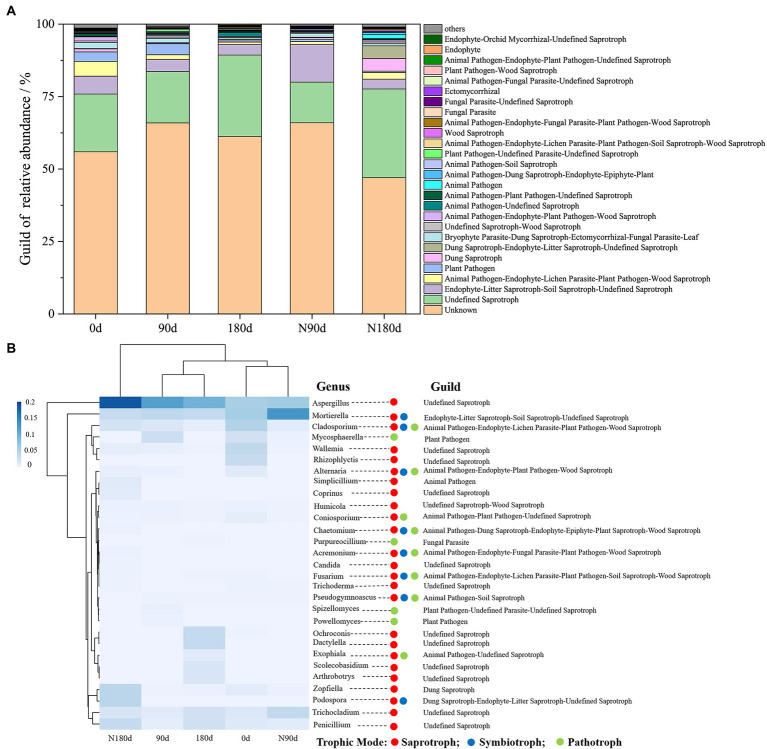
The relative abundance of fungal functional groups (guild) in different samples was annotated using FUNGuild **(A)**; Guild of fungal genera with an average relative abundance greater than 0.01% annotated by FUNGuild **(B)**.

The functional profiles of soil microbial communities could help us to better comprehend the ecological implications of responses to Cr fraction migration and occurrence shifts. The potential functions of bacterial and fungal communities for Cr fraction occurrence and transformation under wet and dry conditions were predicted using PICRUSt2 (Figure 5) and FUNGuild (Figure 6). Among them, genetic information processing and signaling and cellular processes in the protein families [level 2 KEGG orthology (KO)] accounted for the largest proportion of bacterial function. In addition, carbohydrate metabolism, energy metabolism, and amino acid metabolism were the three main metabolic pathways during the whole process. As core resource metabolic pathways, they were potential drivers of soil and river microbial community structure and function (Li et al., 2017c; Ren et al., 2017; He et al., 2020). Thus, the proportions of bacterial community functions (including cell growth, signal transduction, and material metabolism) could remain high under the stress of soil Cr contamination. A previous study summarized microbial metabolic mechanisms for potentially toxic element pollution, including direct export of ions, converting ions to a less poisonous state, and export of ions to the periplasm followed by reduction of ions into a less toxic form (Silver and Phung, 1996; Yan et al., 2020). In addition, as shown by the functions at level 3 KO, we focused on the significant difference of functional categories in metabolism functions between the LP and ND stages. Notably, the abundance of metabolism of terpenoids and polyketides, biosynthesis of other secondary metabolites, and xenobiotics biodegradation and metabolism (level 3 KOs) were significantly higher (proportion > 2.0%, *p* < 0.05) in the LP stage than in the ND stage (Figure 5). Several studies suggested that there was a correlation between the rate of xenobiotics biodegradation and metabolism and the abundance of xenobiotic degradation genes (Kansole and Lin, 2016; Zhang et al., 2020a). Related research reported that the dual effects of potentially toxic element pollution and temperature stimulated the metabolism of certain soil substances, including lipids, terpenoids, and polyketides, as well as xenobiotics biodegradation and biosynthesis of other secondary metabolites (Jia et al., 2020). We speculated that the migration and transformation of Cr fractions in the LP led to the enrichment of terpenoids and polyketides, xenobiotics biodegradation, and biosynthesis of other secondary metabolites.

Little information has been available on the functional response of microbial communities, especially fungal communities, to the migration and occurrence of Cr fractions in soil. A relevant study that annotated bacterial and fungal community functions showed that high Cr contents not only reduced the number of indicator microorganism groups but also significantly decreased the abundances of total functional genes and many metabolic processes (Zeng et al., 2020). Using the FUNGuild database, the relative abundances of the ecological guilds of fungi present in the samples were examined (Figure 6A). Figure 6B shows the 29 genera with predicted functional information and abundances. The abundances of Aspergillus, Mortierella, Cladosporium, and Mycosphaerella were higher in the LP stage than in the ND stage. In addition, the abundances of Penicillium, Trichocladium, Ochroconis, Dactylella, Scolecobasidium, and Arthrobotrys were higher in the ND stage but reduced to <0.01% in the LP stage. The functional genera were annotated in different tropical modes. Given our previous results, Cladosporium was significantly positively correlated with F1, F2, and F3 under LP conditions (Figure 4B), and Cladosporium annotated gene abundance was significantly higher in the LP stage than in the ND stage (Figure 6B). These results indicated that the migration and occurrence of Cr fractions in the LP caused enrichment of functional genes and the metabolic abundance of Animal Pathogen–Endophyte–Lichen Parasite–Plant Pathogen–Wood Saprotrophs in soil. Further studies are needed in Cr-contaminated systems using marker genes and metagenomic sequencing methods to comprehensively assess gene categories of associated microbial groups.

## Conclusion

The four fractions of Cr transformed into one another over time, and F1 and F2, which readily migrate, could be transformed into other relatively stable fractions under dry conditions. The differences in dry and wet environments in soil altered the correlations between nitrogen cycle and Cr fractions, and NH_3_-N actively promoted the transformation of loosely bound F1 and F2 into other more stable fractions. In addition, the conversion between dry and wet conditions altered the relationships between Cr fractions and bacterial and fungal groups. The microbial genera Flavihumibacter, Altererythrobacter, Methylobacillus, Ramlibacter, and Cladosporium were significantly associated with the migration and occurrence of Cr fractions. Moreover, the proliferation of Methylobacillus, Ellin6067, and MND1 was related to Cr fractions during the wet–dry conversion process resulting in the enrichment of terpenoids and polyketides, xenobiotic biodegradation, and biosynthesis of other secondary metabolites, which could alleviate the environmental toxicity of Cr. This study provides comprehensive information and new insights to accurately assess the environmental toxicity of Cr pollution through the dual effects of functional microbes and environmental factors.

## Data availability statement

The datasets presented in this study can be found in online repositories. The names of the repository/repositories and accession number(s) can be found at: https://www.ncbi.nlm.nih.gov/, SRP299378; SRP299488.

## Author contributions

SZ, RY, and JT performed experiments. HL, SZ, RY, JT, XL, LJ, BM, and ES contributed to conception and design of the study. SZ, HL, and RY analyzed and interpreted data. SZ and HL wrote the first draft of the manuscript. All authors contributed to the article and approved the submitted version.

## Funding

This research was funded by the National Key R&D Program of China (2018YFC1801804) and Chinese National Natural Science Foundation (51504298).

## Conflict of interest

The authors declare that the research was conducted in the absence of any commercial or financial relationships that could be construed as a potential conflict of interest.

## Publisher’s note

All claims expressed in this article are solely those of the authors and do not necessarily represent those of their affiliated organizations, or those of the publisher, the editors and the reviewers. Any product that may be evaluated in this article, or claim that may be made by its manufacturer, is not guaranteed or endorsed by the publisher.

## Footnotes

^1^http://rdp.cme.msu.edu/classifier/classifier.jsp.

^2^http://huttenhower.sph.harvard.edu/galaxy (Accessed July 27, 2020).

^3^http://www.stbates.org/guilds/app.php (Accessed July 27, 2020).
