# Supplementary material for: Insight into functional microorganisms in wet–dry conversion to alleviate the toxicity of chromium fractions in red soil
Source: Front Microbiol. 2022 Aug 10;13:977171. doi: 10.3389/fmicb.2022.977171 (PMC9399814; doi:10.3389/fmicb.2022.977171)
Supplement: Supplementary file 1 [file Data_Sheet_1.docx]

Supplementary Material

# Supplementary Figures


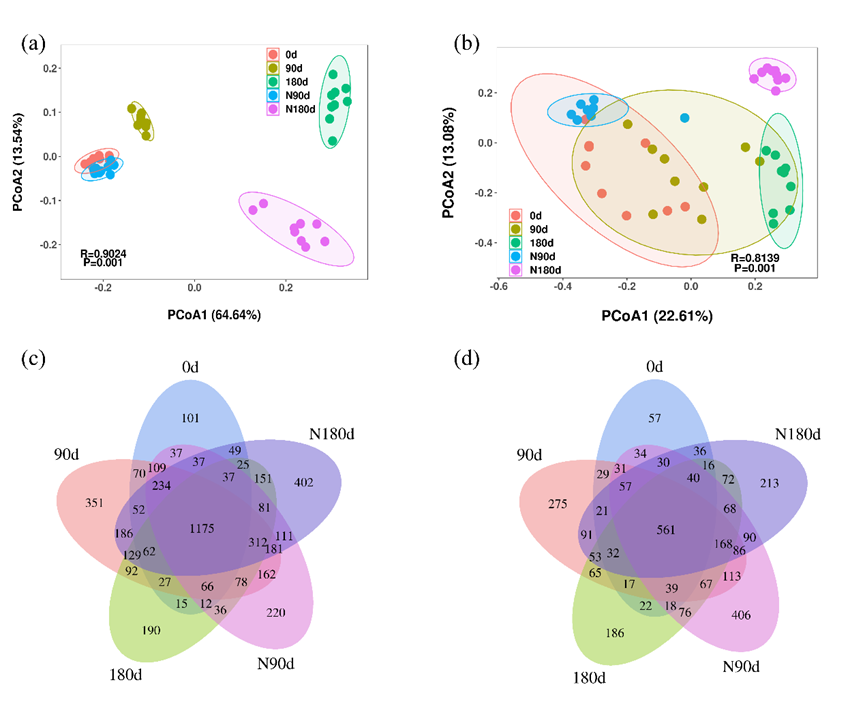


**Fig. S1.** Analysis of the bacterial (a) and fungal (b) communities in soil by principal co-ordinates analysis (PCoA) at wet and dry stages. Each point represents the individual microbial community in soils. Analysis of the bacterial (c) and fungal (d) communities in soil by Venn diagram at different times. Data are presented as means ± SD (n = 9). N90d: 90 days of control group; N180d: 180 days of control group.


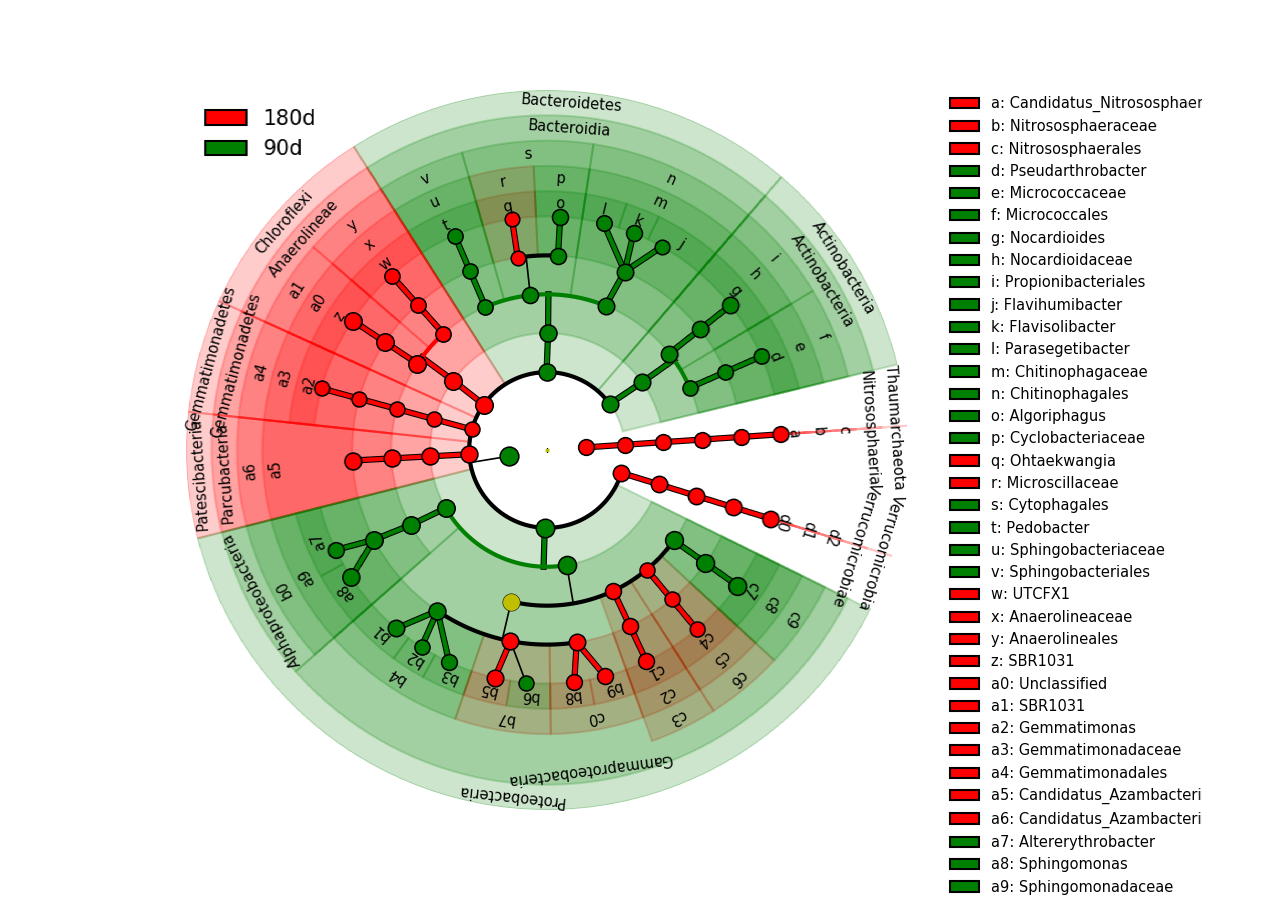


**Fig. S2.** LEfSe analysis of bacterial community samples between 90d and 180d treatment (golden circles mean non-significant difference (*p* > 0.05) in abundance among different soil samples; other colors mean biomarkers with significant differences (*p* < 0.05) in different times).





**Fig. S3.** The Ordination plot from redundancy analysis (RDA) showing the relationship between the microbial community structures and environmental variables. Each point represents the individual microbial community in soils. Arrow direction indicates the correlation among soil properties; arrow length indicates the strength of the correlation. ORP: oxidation reduction potential; TN: total N; NH3: ammonium nitrogen; NO3: Nitrate nitrogen; TK: total K; OK: available K, F1: acid-soluble Cr; F2: reducible Cr; F3: oxidizable Cr; F4: residual Cr.


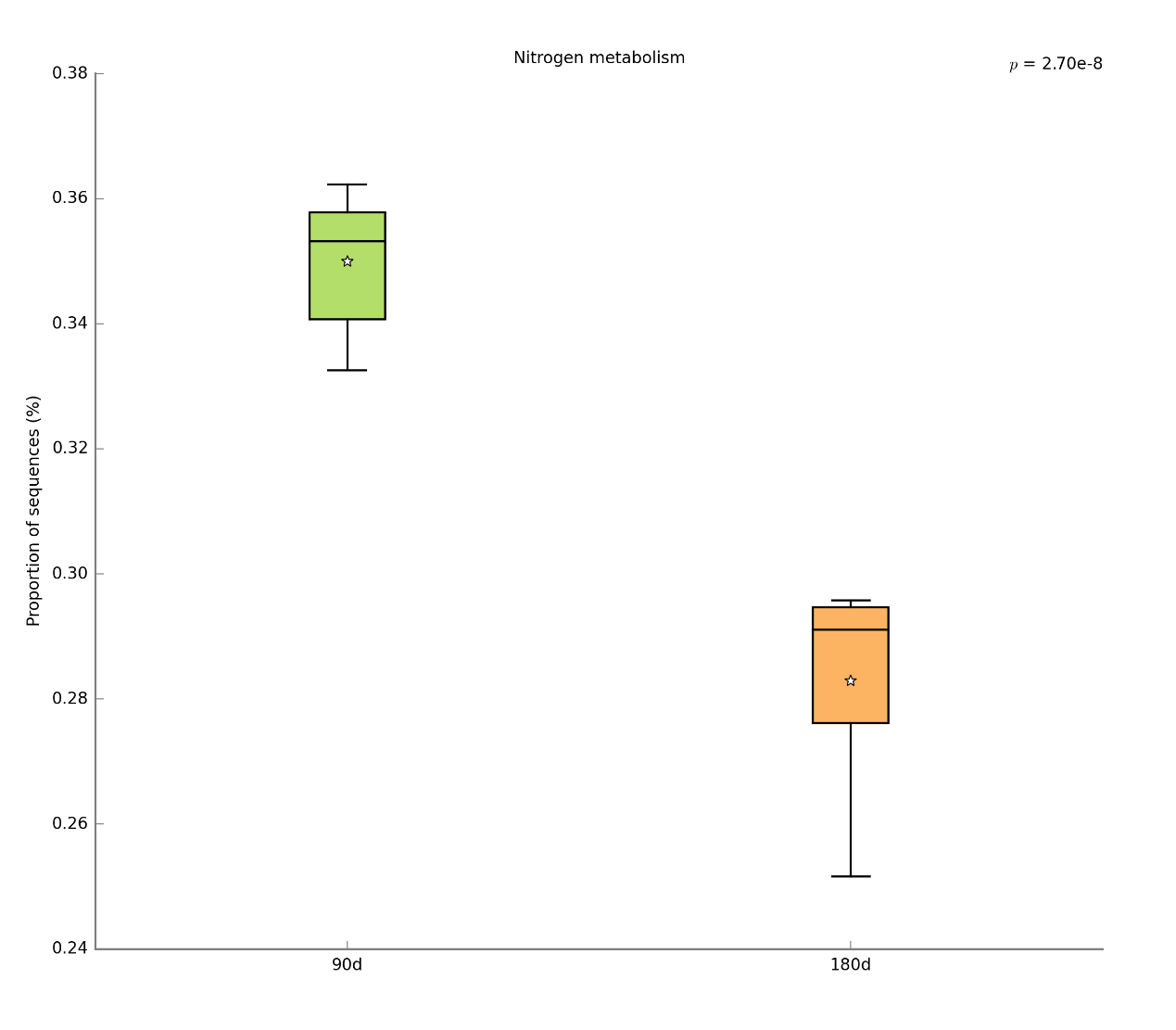


**Fig. S4.** Box plot analysis of relative abundance of nitrogen metabolism genes.


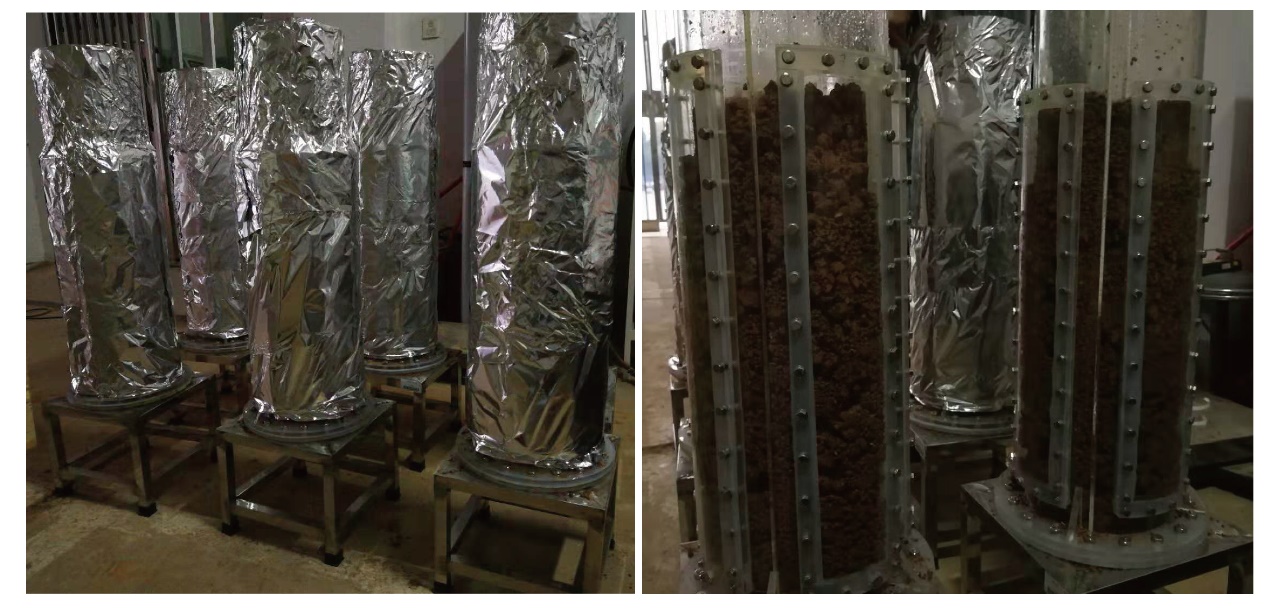

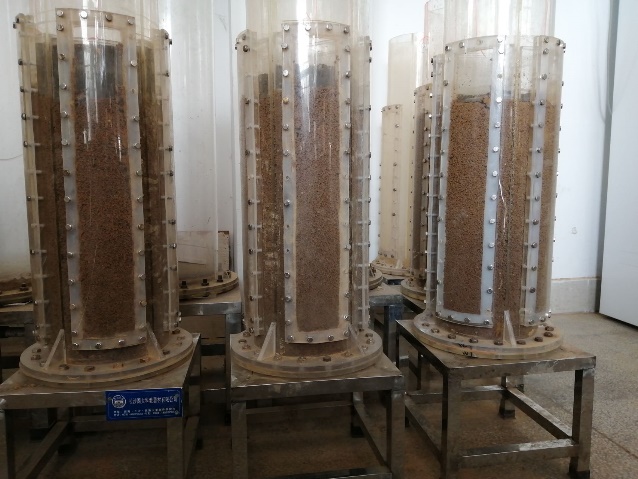


**Fig. S5.** Polymethyl methacrylate (PMMA) columns.

# Supplementary Tables

**Table S1** Soil physicochemical properties (means ± SD, n = 3) with time and space distribution

| Properties | OS | layer | CK | Time | | |
| --- | --- | --- | --- | --- | --- | --- |
|  | 0d |  |  | 30d | 90d | 180d |
| OM (%) | 0.16±0.08 | G | 0 | 0.4 ±0.02a | 0.16±0.03b | 0.11±0.013b |
|  |  | T | 0.7±0.13 | 0.98±0.25a | 0.17±0.08b | 0.21±0.047b |
|  |  | M | 0.4±0.03 | 0.39±0.05a | 0.13±0.04b | 0.4±0.12a |
|  |  | S | 0.4±0.11 | 0.27±0.03b | 0.26±0.13b | 0.34±0.022a |
| TN (mg/Kg) | 369.50 ±44.31 | G | 0 | 1467.7±542.1b | 1715.5±343.2a | 1202.43±331b |
|  |  | T | 421.4±16.7 | 575.5±58.7b | 651.6±22.3a | 568.4±25.6b |
|  |  | M | 498.1±51.7 | 444.1±39.0b | 476.0±28.6b | 534.55±47a |
|  |  | S | 538.1±15.6 | 558.8±19.9a | 369.6±27.6b | 409.96±33.8c |
| TP (mg/Kg) | 74.52 ±5.31 | G | 0 | 91.5±22.3b | 168.8 ±42.3a | 107.07±16.2b |
|  |  | T | 109.2±2.0 | 109.0 ±2.3b | 153.3±4.8a | 132±19.9c |
|  |  | M | 126.4±3.5 | 325.6±12.0a | 104.0±3.2b | 135.24±31.2b |
|  |  | S | 142.8±4.3 | 121.5±9.6a | 87±6.3b | 93.8±7.9b |
| AP (mg/Kg) | 67.16 ±7.31 | G | 0 | 22.36±5.3b | 28.54 ±0.3a | 22.34±2.8b |
|  |  | T | 24.2±4.4 | 25.56±5.5a | 28.59±3.3a | 23.85±4.7a |
|  |  | M | 24.0±5.4 | 23.18±6.3a | 27.2±2.3a | 24.08±5.3a |
|  |  | S | 26.7±0.9 | 24.2±6.2a | 28.55±5.2a | 26.64±3.7a |
| NH_3_-N (mg/Kg) | 4.45±0.66 | G | 0 | 9.66 ±0.3b | 13.18±0.6a | 6.65±0.21c |
|  |  | T | 5.2±1.5 | 8.4±0.6b | 8.8±1.6b | 13.79±2.6a |
|  |  | M | 5.2±1.4 | 5.7±0.2b | 16.6±3.6a | 15.06±4.2a |
|  |  | S | 4.6±0.9 | 4.8±0.3b | 16.1±4.8a | 4.85±0.34b |
| NO_3_-N (mg/Kg) | 25.27 ±3.65 | G | 0 | 180.5 ±3.3a | 97.94 ±0.4b | 96.03±8.8b |
|  |  | T | 26.2±2.1 | 36.6±2.2a | 31.6±1.1b | 31.05±3.9b |
|  |  | M | 27.0±3.1 | 28.1±3.8a | 29.4±4.2a | 27.83±6.5a |
|  |  | S | 24.6±3.4 | 25.4±2.8a | 27.4±3.6a | 22.4±4.1a |
| TK (g/Kg) | 18.17±2.56 | G | 0 | 11.16 ±0.3b | 15.31 ±0.6a | 9.65±0.8b |
|  |  | T | 14.2±2.2 | 13.9±4.2b | 13.5±1.2b | 19.01±2.2a |
|  |  | M | 13.1±0.5 | 13.3±0.2b | 13.2±1.9b | 19.72±1.3a |
|  |  | S | 14.4±0.5 | 13.5±1.1b | 12.9±3.4b | 19.69±4.1a |
| OK (mg/Kg) | 105.67±4.48 | G | 0 | 43.13±6.3b | 63.14 ±5.9a | 61.04±4.8a |
|  |  | T | 112.5±3.0 | 105.3±5.8b | 114.6±2.8a | 113.44±6.9a |
|  |  | M | 112.4±9.5 | 113.9±4.9a | 113.2±3.3a | 120.87±7.1a |
|  |  | S | 107.7±7.4 | 113.5±6.6a | 111.3±3.8a | 114.19±5.2a |
| pH | 7.38±0.47 | G | 0 | 8.2±0.2a | 8.15±0.2a | 8.23±0.9a |
|  |  | T | 7.6±0.07 | 7.9±0.04a | 7.9±0.07a | 7.84±0.04b |
|  |  | M | 7.6±0.06 | 7.7±0.07a | 7.7±0.03a | 7.51±0.06b |
|  |  | S | 7.5±0.03 | 7.7±0.02a | 7.6±0.09a | 7.45±0.07b |
| ORP | 206.73 ±0.95 | G | 0 | 202.3 ±3.6c | 211.35 ±2.3b | 215.8±3.7a |
|  |  | T | 235.7±2.1 | 177.4±6.9c | 188.9±4.7b | 198.73±3.4a |
|  |  | M | 225.0±6.9 | 207.1±2.2a | 195.3±4.2b | 202.77±3.5a |
|  |  | S | 219.2±4.1 | 197.1±0.3b | 199±2.6b | 213.67±6.9a |

**Note：**Different lowercase letters in same row indicated significant difference (*P*< 0.05, LSD) among different groups. ORP: oxidation reduction potential; OM: soil organic matter; TN: total N; TP: total P; AP: available P; NH_3_-N: ammonium nitrogen; NO_3_-N: Nitrate nitrogen; TK: total K; OK: available K; OS: original soil; CK: control group; G: Cr slag layer; T: top layer; M: middle layer; S: substratum layer.

**Table S2** Alpha diversity of different layers in wet and dry stage.

|  | alpha diversity | CK | T | M | S |
| --- | --- | --- | --- | --- | --- |
| 90d | observed_species | 1111.1±65.3b | 1277±38.3a | 1308±45.1a | 1291.3±72.2a |
|  | Shannon | 4.52±0.12b | 5.08±0.058a | 4.92±0.059a | 4.78±0.098ab |
|  | Simpson_evenness | 0.64±0.014b | 0.70±0.008a | 0.68±0.009a | 0.66±0.013ab |
| 180d | observed_species | 1396.2±88.7a | 944.6±35.1b | 944.6±22.8b | 1040±55.6c |
|  | Shannon | 5.49±0.14a | 4.66±0.12b | 4.71±0.13b | 4.84±0.49b |
|  | Simpson_evenness | 0.75±0.014a | 0.68±0.014b | 0.68±0.015b | 0.69±0.055b |

**Table S3** Alpha diversity between with bacterial and fungal communities in different time periods.

|  | alpha diversity | 0d | 90d | 180d | N90d | N180d |
| --- | --- | --- | --- | --- | --- | --- |
| bacterial | observed_species | 1244±52.1ab | 1310.2±33.1a | 1111.1±41.2b | 976.4±52.3c | 1396.2±66.8a |
|  | Shannon | 4.71±0.21bc | 4.93±0.03b | 4.74±0.15bc | 4.53±0.10c | 5.49±0.03a |
|  | Simpson_evenness | 0.028±0.003c | 0.027±0.003c | 0.039±0.004b | 0.026±0.002c | 0.053±0.004a |
| fungal | observed_species | 584.3±18.6a | 526.8±15.9b | 585.8±34.9a | 481.7±22.7c | 548.2±32.5ab |
|  | Shannon | 5.48±0.52a | 4.79±0.49b | 3.84±0.88c | 4.86±0.71b | 5.16±0.39ab |
|  | Simpson_evenness | 0.92±0.035a | 0.89±0.05b | 0.72±0.067c | 0.89±0.057b | 0.91±0.042a |
